# The Circadian Rhythm Gene Network Could Distinguish Molecular Profile and Prognosis for Glioblastoma

**DOI:** 10.3390/ijms26125873

**Published:** 2025-06-19

**Authors:** Fangzhu Wan, Zongpu Zhang, Jinsen Zhang, Jiyi Hu, Weixu Hu, Jing Gao, Minjie Fu, Yuan Feng, Lin Kong

**Affiliations:** 1 Department of Radiation Oncology, Shanghai Proton and Heavy Ion Center, Fudan University Shanghai Cancer Center, Shanghai 201321, China; wanofficial@foxmail.com (F.W.);; 2 Shanghai Key Laboratory of Radiation Oncology, Shanghai 201321, China; 3 Shanghai Engineering Research Center of Proton and Heavy Ion Radiation Therapy, Shanghai 201321, China; 4 Department of Thoracic Surgery and State Key Laboratory of Genetics and Development of Complex Phenotypes, Fudan University Shanghai Cancer Center, Shanghai 200032, China; 5 Institute of Thoracic Oncology, Fudan University, Shanghai 200032, China; 6 Department of Oncology, Shanghai Medical College, Fudan University, Shanghai 200032, China; 7 Department of Neurosurgery, Huashan Hospital, Shanghai Medical College, Fudan University, Shanghai 200040, China; 8 Neurosurgical Institute of Fudan University, Shanghai 200040, China; 9 Shanghai Clinical Medical Center of Neurosurgery, Shanghai 200040, China; 10 Shanghai Key Laboratory of Brain Function and Restoration and Neural Regeneration, Shanghai 200040, China; 11 Department of Radiation Oncology, Shanghai Proton and Heavy Ion Center, Shanghai 201321, China

**Keywords:** glioblastoma, circadian rhythm, unsupervised cluster analysis, LASSO, proneural subtype

## Abstract

Increasing evidence highlights the role of aberrant circadian rhythm gene expression in glioblastoma (GBM) progression, but the impact of the circadian rhythm gene network on GBM molecular profiles and prognosis remains unclear. A total of 1042 GBM samples from six public datasets, TCGA and CGGA, were analyzed, with GBM samples stratified into three circadian core-gene patterns using unsupervised clustering based on the expression profiles of 17 circadian rhythm genes. The Limma R package identified differentially expressed genes (DEGs) among the three patterns, and a secondary clustering system, termed circadian-related gene pattern, was established based on DEGs. A circadian risk score was constructed using the Least Absolute Shrinkage and Selection Operator (LASSO) regression algorithm, and the efficiency of these patterns and the circadian risk score in distinguishing molecular profiles and predicting prognosis was systematically analyzed. The relationship between the circadian risk score and response to immune or targeted therapy was examined using the GSE78200 and IMvigor210 datasets. The results showed that GBM patients were clustered into three circadian core-gene patterns based on the expression profiles of 17 core circadian genes, with distinct molecular profiles, malignant characteristics, and patient prognoses among the patterns. Thirty-two DEGs among these patterns were identified and termed circadian-related genes, and secondary clustering based on these 32 DEGs classified GBM samples into two circadian-related gene patterns, which also predicted molecular profiles and prognosis. A circadian risk scoring system was established, allowing the calculation of individual risk scores based on the expression of 10 genes, where GBM patients with lower circadian risk scores had prolonged overall survival and less aggressive molecular subtypes, while higher circadian risk scores correlated with better responses to MAPK-targeted therapy. In conclusion, this study established two clustering patterns based on 17 circadian rhythm genes or 32 circadian-related genes, enabling the rapid classification of GBM patients with distinct molecular profiles and prognoses, while the circadian risk scoring system effectively predicted survival, molecular profiles, and therapeutic responses for individual GBM patients, demonstrating that the circadian rhythm gene network can distinguish molecular profiles and prognosis in GBM.

## 1. Introduction

Glioblastoma (GBM) is the most common primary malignant brain tumor, with a median survival of approximately 15 months despite aggressive multimodal therapy [1]. The 2021 WHO CNS5 classification of brain tumors has updated the diagnosis and definition of GBM by integrating molecular features such as IDH wild type, EGFR amplification, TERT promoter mutation, and CDKN2A/2B deletion, highlighting the predominant role of endogenous biological events in GBM diagnosis [2]. To identify homogeneous subcategories, GBM has been classified into several subtypes (Mesenchymal [MES], Classical [CL], and Proneural [PN]) based on transcriptional features proposed by Verhaak and colleagues [3,4]. However, this transcriptome-based profiling of GBM is neither economical nor easily applicable in clinical settings, necessitating the development of alternative solutions based on a more restricted collection of genes.

Circadian rhythm is an endogenous biological event that is regulated by a restricted collection of genes [5]. This endogenous timing program controls a wide range of downstream effector genes. The four major genetic drivers for GBM newly proposed in the WHO CNS5 classification—EGFR, PDGFRA, CDK4, and NF1—are closely regulated by the circadian network [6]. Additionally, circadian rhythm gene networks control fundamental characteristics of GBM, such as cancer cell stemness [7], hypoxic conditions [8], and the immune microenvironment [9,10]. These characteristics contribute to GBM’s self-renewal capability, anaerobic metabolism, and defective anti-tumor immunity [11,12]. Numerous efforts have been made to target circadian rhythm genes for GBM therapy [13,14]. However, translating these findings into clinical applications remains challenging, partly due to the divergent expression patterns of circadian rhythm gene networks among patients. Thus, a comprehensive model to subtype GBM based on distinct circadian rhythm gene expression patterns is imperative.

In this study, we constructed two patterns for GBM subtyping based on 17 circadian rhythm genes and 32 circadian-related genes, respectively, which enabled rapid subtyping of GBM patients with distinct molecular profiles and overall survival outcomes. Additionally, a circadian risk scoring system was constructed based on 10 circadian-related genes, which effectively predicted survival, molecular profile and therapeutic responses for individual GBM patients. These findings suggest that the circadian rhythm gene network could distinguish molecular profile and prognosis for GBM and might provide economical and easily applicable predicting models for GBM patients in clinical applications.

## 2. Results

### 2.1. Landscape of Circadian Rhythm Gene Expression in GBM

**Figure 1:**
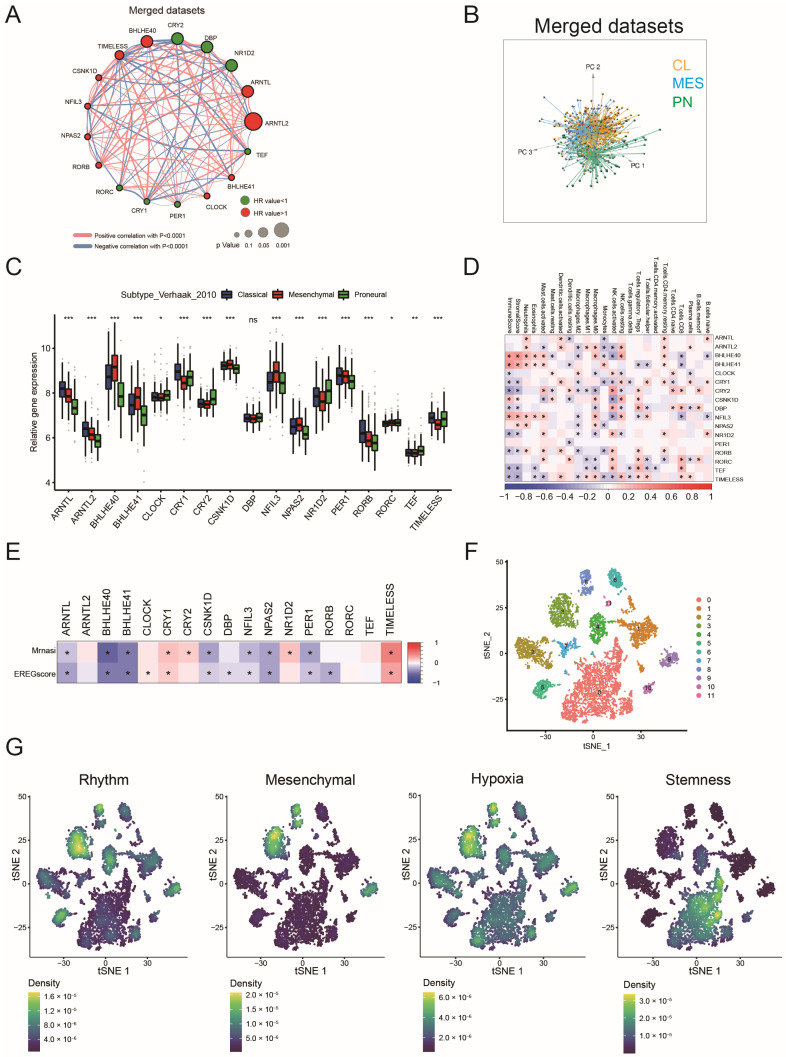
Landscape of genetic and expression variation of circadian rhythm genes in GBM: (**A**) The interaction between circadian rhythm genes in GBM cases from merged dataset (MDSet). The circadian rhythm genes with HR values <1 and >1 are depicted by circles in green and red, respectively. The lines connecting circadian rhythm genes represented their interaction with each other. The size of each circle represented the *p* value for HR value; (**B**) Principal component analysis (PCA) for the expression profiles of 17 circadian rhythm genes to distinguish CL, PN, MES GBM in MDSet cohort. Three subgroups were identified, indicating the CL, PN, MES GBM were well distinguished based on the expression profiles of circadian rhythm genes. CL, PN, MES GBM are marked with yellow, blue and green, respectively; (**C**) The relative gene expression of 17 circadian rhythm genes in CL, PN, MES GBM (Kruskal–Wallis test). Data were retrieved from MDSet cohort; (**D**) Correlation between 17 circadian rhythm genes and immune/stromal cell infiltration in MDSet cohort. Data were evaluated by Spearman analysis; (**E**) Correlation between 17 circadian rhythm genes and stemness indices in MDSet cohort. Data were evaluated by Spearman analysis; (**F**) Single-cell RNA sequencing of GSE138794 visualizing tSNE cell clusters; (**G**) Single-cell RNA sequencing visualizing the enrichment scores of the 17-gene-based circadian rhythm signature, the Verhaak_GBM_MES signature, hypoxia signature and stemness signature. * *p* < 0.05, ** *p* < 0.01, *** *p* < 0.001.

Seventeen circadian rhythm genes were referenced from the previous study [15]. Pearson correlation analysis showed the seventeen genes could be identified in a cluster, indicating a synchronous function (Figure 1A and Supplementary Figure S1A,B). Three genes (ARNTL, ARNTL2, BHLHE40) from MDSet (six merged datasets) could represent unfavorable prognostic factors for GBM, whereas CRY2, DBP and NR1D2 were associated with better prognosis (Figure 1A). Additionally, principal component analysis (PCA) confirmed that these seventeen genes could distinguish between the MES, PN, and CL subtypes in both the MDSet and TCGA cohort (Figure 1B and Figure S1C). Most circadian genes exhibited subtype-specific expressions, such as BHLHE40, BHLHE41, and NFIL3 in MES; TEF and NR1D2 in PN; and ARNTL, ARNTL2, CRY1, PER1, and RORB in CL subtype (Figure 1C and Figure S1D).

Thirteen of the seventeen circadian genes were associated with immune/stromal cell infiltration in GBM (Figure 1D and Figure S1E). Moreover, most circadian rhythm genes showed a correlation with cell stemness, with nine genes negatively correlated with the stemness index and five genes showing a positive association (Figure 1E). Single-cell analysis showed that cell clusters with a high density of circadian rhythm signatures (clusters 3 and 8 in Figure 1F) tended to exhibit more MES and hypoxia signatures but fewer stemness characteristics, indicating that circadian rhythm genes might be an origin for diverse molecular profiles in GBM (Figure 1F,G).

Despite the occurrence of mutation among 17 circadian rhythm genes, the mutation frequency of these genes was rather low (<1%), suggesting the high conservation of circadian rhythm genes (Supplementary Figure S1F,G). Copy number variation (CNV) analysis demonstrated the highest CNV amplification or deletion frequency for CLOCK and DBP, respectively. In summary, these 17 circadian rhythm genes are significant contributors to the molecular profiles and prognostic outcomes in GBM.

### 2.2. Circadian Core-Gene Patterns and Circadian-Related Gene Patterns Could Stratify GBM Patients According to Molecular Profile and Prognosis

**Figure 2:**
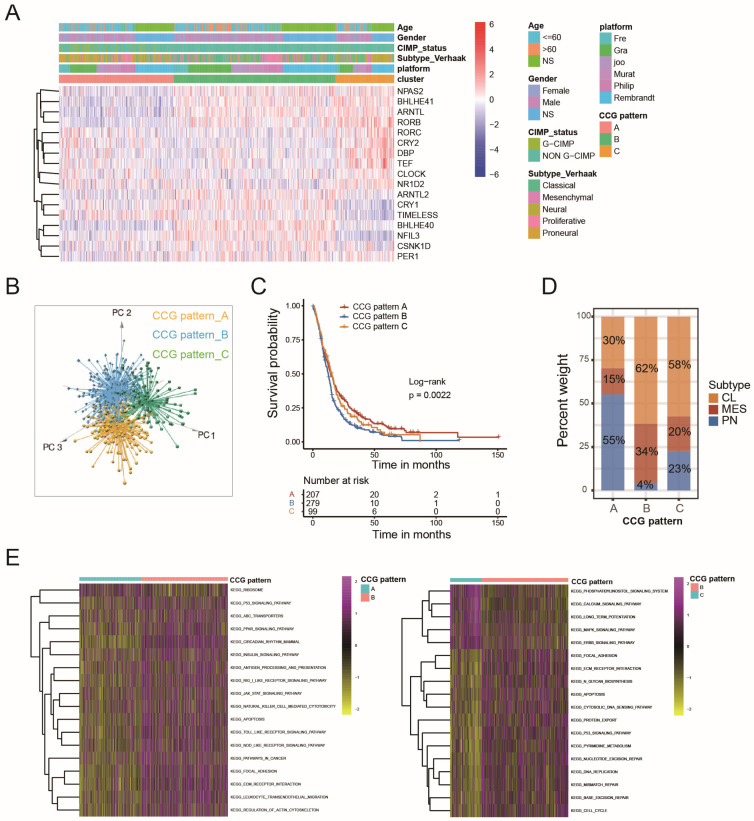
Circadian core-gene patterns and transcriptomic features of each pattern: (**A**) Unsupervised clustering of 17 circadian rhythm genes in MDSet cohort resulted in three circadian core-gene patterns. Red represents the relatively high expression of circadian rhythm genes and blue represents the relatively low expression; (**B**) PCA for the transcriptome profiles of three circadian core-gene patterns, showing a remarkable difference in transcriptome between different circadian core-gene patterns; (**C**) Kaplan–Meier curves indicated circadian core-gene patterns were markedly related to overall survival of patients in MDSet cohort, of which 230 cases were classified as Pattern A, 290 cases as Pattern B and 105 cases as Pattern C (*p* = 0.0022, Log-rank test); (**D**) The proportion of transcriptome subtypes in the three circadian core-gene patterns. CL subtype, yellow; MES subtype, red; PN subtype, blue; (**E**) GSVA enrichment analysis showing the activation states of biological pathways in distinct circadian core-gene patterns. The heatmap was used to visualize these biological processes, and pink represented activated pathways and yellow represented inhibited pathways. Left, circadian core-gene Pattern A vs. B; right, circadian core-gene Pattern B vs. C.

Three distinct circadian core-gene patterns (CCG Patterns A, B, and C) were identified in GBMs from MDSet using unsupervised clustering based on transcriptome profiles of seventeen circadian rhythm core genes (Figure 2A). PCA confirmed the discrimination among these three patterns (Figure 2B). The overall survival differed significantly between patients with different patterns: CCG Pattern A displayed the most favorable prognosis, while CCG Pattern B showed the most adverse (Figure 2C). This variance in prognosis can be partially attributed to the distribution of molecular subtypes within each pattern. Fifty-five percent of samples in CCG Pattern A displayed a molecular profile similar to the PN subtype, whereas the CL subtype was predominant in CCG Patterns B (62%) and C (58%) (Figure 2D). Notably, the pattern with the worst prognosis (CCG Pattern B) contained the highest percentage of the MES subtype (34%) and the lowest percentage of the PN subtype (4%), in contrast to the best prognostic pattern (CCG Pattern A) (Figure 2D). The AUC for circadian core-gene patterns in diagnosing the PN subtype reached 0.76 (Supplementary Figure S2A,B). Additionally, 100% of cases in CCG Pattern B were non-G-CIMP, while 24% of patients in CCG Pattern A were G-CIMP positive (Supplementary Figure S2C).

Considering gender differences in circadian rhythms, the male and female proportions in CCG Patterns A, B and C were compared and no significant difference was found in gender composition between these three CCG patterns (Supplementary Figure S2D). In addition, male and female patients in MDSet were stratified separately by unsupervised clustering based on transcriptome profiles of seventeen circadian rhythm core genes (Supplementary Figure S2E for males and Supplementary Figure S2F for females). Similarly, three patterns displaying different overall survival could be discriminated among male and female patients (Supplementary Figure S2G for males and Supplementary Figure S2H for females). In accordance with the results for MDSet in whole, the pattern with the worst prognosis in both male and female subgroups (Pattern B for males, Pattern B for females) contained the highest percentage of the MES subtype (34% for males and 37% for females) and a relatively lower percentage of the PN subtype (6% for males and 14% for females, Supplementary Figure S2I for males and Supplementary Figure S2J for females).

Molecular pathway analysis using GSVA showed that tumors in CCG Pattern A were positively correlated with protein translation (ribosome), CCG Pattern B tumors were characterized by tumorigenesis (P53 signaling pathway) and immune response (PPAR signaling pathway), and CCG Pattern C was mainly associated with the cell cycle, DNA repair, and metabolism (glycan biosynthesis, pyrimidine metabolism) (Figure 2E). These findings suggest that circadian core-gene patterns can distinguish different molecular profiles, particularly the PN subtype, thereby demonstrating prognostic value in GBM.

**Figure 3:**
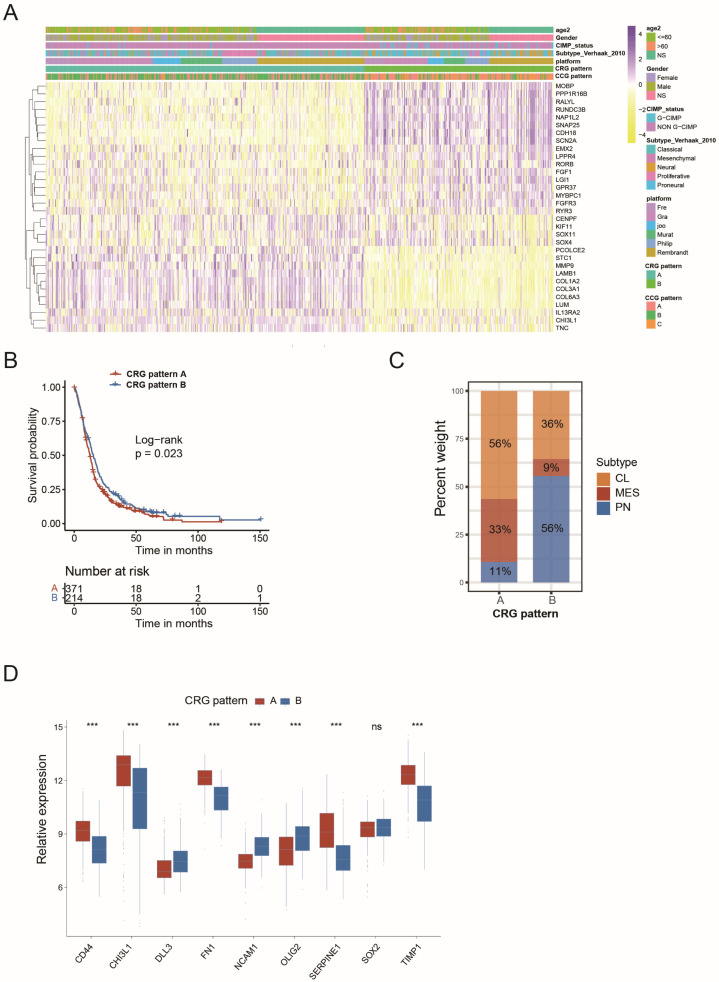
Generation of circadian-related gene patterns and exploration of molecular profiles: (**A**) Unsupervised clustering of overlapping circadian rhythm-related genes in MDSet cohort to classify patients into different genomic subtypes, termed as circadian-related gene Patterns A and B. The circadian-related gene patterns, circadian core-gene patterns, Verhaak molecular subtypes, G-CIMP status and age were used as patient annotations; (**B**) Kaplan–Meier curves indicated circadian-related gene patterns were markedly related to overall survival of 585 patients in MDSet cohort, of which 371 cases were classified as circadian-related gene pattern A, 214 cases as circadian-related gene pattern B (*p* = 0.023, Log-rank test); (**C**) The proportion of transcriptome subtypes in the two circadian-related gene patterns. CL subtype, yellow; MES subtype, red; PN subtype, blue; (**D**) The PN and MES markers in circadian-related gene Patterns A and B (*** *p* < 0.001 by Kruskal–Wallis test; ns, not significant).

To further characterize the functional roles of the three circadian core-gene patterns, we identified differentially expressed genes (DEGs) between the patterns using the Limma R package, resulting in 32 DEGs that were differentially expressed among CCG Patterns A, B, and C (Supplementary Figure S3A). These genes were identified as circadian rhythm-related genes. Unsupervised clustering analysis based on these 32 genes classified GBM samples into two genomic subtypes: circadian-related gene Patterns A and B (CRG Patterns A and B; Figure 3A). This novel subtyping model effectively discriminated prognosis, as patients in CRG Pattern A had shorter overall survival compared to those in CRG Pattern B (*p* = 0.023, Figure 3B). In the meantime, the two CRG patterns also exhibited distinct molecular profiles. The PN subtype accounted for 11% of CRG Pattern A compared to 56% in CRG Pattern B (Figure 3C). PN marker genes (DLL3, NCAM1, OLIG2) were preferentially expressed in CRG Pattern B, whereas MES gene markers (CD44, CHI3L1, FN1, SERPINE1, TIMP1) were enriched in CRG Pattern A (Figure 3D). Similar to circadian core-gene patterns, the circadian-related gene patterns were also efficient in predicting the PN subtype (AUC = 0.76, Supplementary Figure S3B,C). Additionally, other molecular features associated with better prognosis, such as G-CIMP, were less frequently found in CRG Pattern A (Supplementary Figure S3D). These results suggest that the circadian rhythm gene networks, including circadian core genes and circadian-related genes, enable the stratification of GBM patients according to their molecular profile and prognosis.

### 2.3. Circadian Risk Score Could Show Its Prognostic Value in GBM

**Figure 4:**
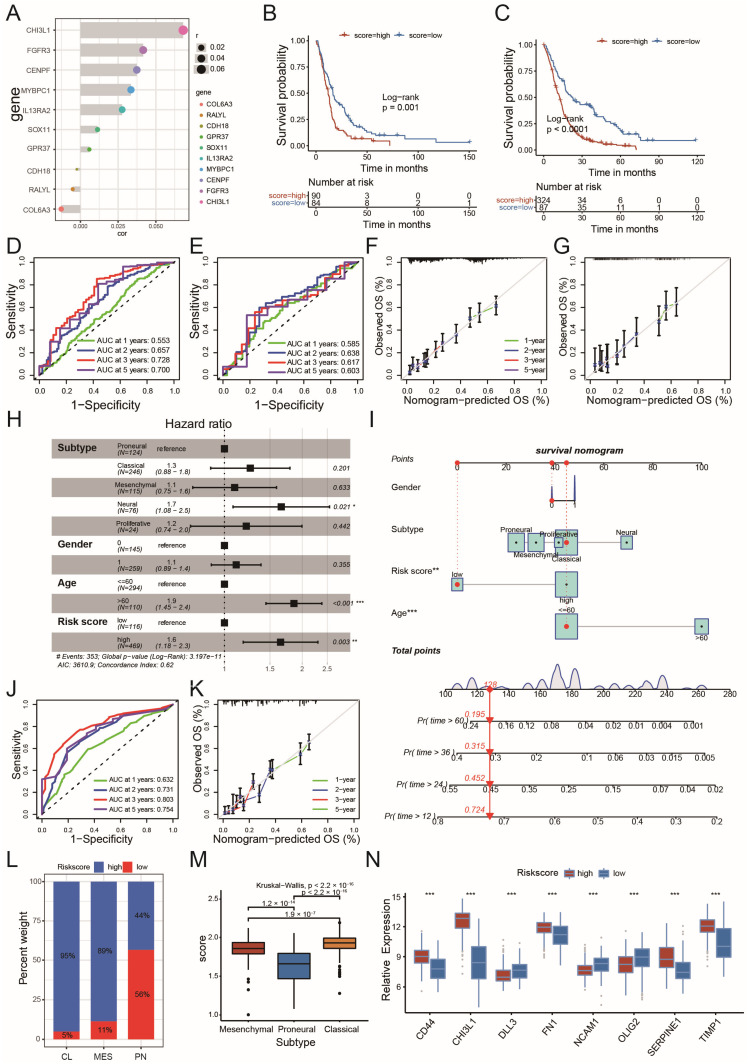
Construction of circadian risk score and evaluation of its predictive potentiality: (**A**) ten DEGs selected by LASSO machine learning methods for scoring system establishment; (**B**) survival analyses for circadian risk score high/low groups in the randomly selected training set from MDSet cohort including 364 cases in the circadian high-risk-score group and 134 cases in the circadian low-risk-score group (Log-Rank test, *p* < 0.0001); (**C**) survival analyses for circadian risk score high/low groups in the randomly selected test set from MDSet cohort including 93 cases in the circadian high-risk-score group and 95 cases in the circadian low-risk-score group (Log-Rank test, *p* = 0.001); (**D**) receiver operating characteristic (ROC) curve for 1, 2, 3, 5-year survival prediction in training set from MDSet cohort. The accuracy was equal to the area under the ROC curves (AUC); (**E**) receiver operating characteristic (ROC) curve for 1, 2, 3, 5-year survival prediction in test set from MDSet cohort. The accuracy was equal to the area under the ROC curves (AUC); (**F**) calibration curves for 1, 2, 3, 5-year survival prediction in training set from MDSet cohort; (**G**) calibration curves for 1, 2, 3, 5-year survival prediction in test set from MDSet cohort; (**H**) multivariate Cox regression model analysis of clinicopathological characteristics and circadian risk score with overall survival in the MDSet cohort; (**I**) nomograms for predicting the probability of patient mortality based on circadian risk score, gender, subtype and age; (**J**,**K**) plots depicted the ROC curve (**J**) and calibration (**K**) of nomograms; (**L**) distribution of circadian risk score high/low cases in CL, PN, MES GBM from the MDSet cohort; (**M**) differences in circadian risk score among CL, PN and MES GBM from the MDSet cohort (Kruskal–Wallis test); (**N**) expression of MES and PN marker genes between circadian risk score high and low group from the MDSet cohort (* *p* < 0.05, ** *p* < 0.01, *** *p* < 0.001 by Kruskal–Wallis test).

To accurately evaluate the prognosis of individual GBM patients, 10 genes were selected from the previously identified DEGs (Supplementary Figure S3A) using the LASSO machine learning method (Figure 4A and Supplementary Figure S4A). A circadian risk scoring scheme was developed based on these genes (Figure 4A and Figure S4A). This risk score effectively stratified GBM patients into long and short survival cohorts (Figure 4B,C; Figure 4B: Training set, Figure 4C: Internal test set). The AUC values for 1, 2, 3, and 5-year survival validated the predictive efficiency of the risk score in both the training (Figure 4D) and internal test sets (Figure 4E). The agreement between risk score predictions and actual observations at 1, 2, 3, and 5-year survival probabilities was excellent after calibration (Figure 4F,G; Figure 4F: Training set, Figure 4G: Internal test set). The risk score was determined to be an independent prognostic factor among other clinicopathological characteristics, with a hazard ratio of 1.6 (Figure 4H). A nomogram demonstrated that a high circadian risk score, along with age, was a key factor in predicting patient mortality (Figure 4I). According to the ROC curve, the nomogram scoring system enabled accurate prediction of patient survival at 1, 2, 3, and 5 years (Figure 4J). This prediction was well-calibrated in the MDSet cohort (Figure 4K). To further verify the prognostic potential of the risk score in external test sets, we examined the system in both the TCGA and CGGA cohorts. Both the risk score and risk score-based nomogram scores were able to predict survival for GBM patients effectively (TCGA: Supplementary Figure S4B–H; CGGA: Supplementary Figure S4I–O). To examine the association of risk score and GBM survival for different genders, both the TCGA and the CGGA cohorts were subdivided according to patients’ sex. The high-risk score patients displayed significantly shorter overall survival in female or male subgroups, indicating the risk scoring system is applicable for all genders (TCGA male: Supplementary Figure S4P; TCGA female: Supplementary Figure S4Q; CGGA male: Supplementary Figure S4R; CGGA female: Supplementary Figure S4S).

In terms of molecular profiles, a low-risk score was predominantly found in the PN subtype, with 56% of PN GBMs presenting a low-risk score. In contrast, high-risk scores were prevalent in the CL and MES subtypes, accounting for 95% and 89%, respectively (Figure 4L,M and Figure S5A). The ROC curve for subtype prediction suggested that the scoring system was particularly efficient for predicting the PN subtype (AUC = 0.85, Supplementary Figure S5B,C). When taking the genders into account, there was still a tendency for high- and low-risk scores to be prevalent in the PN and the CL/MES subtypes, respectively (Supplementary Figure S5D for males and Supplementary Figure S5E for females). In addition, the PN subtype proportion was higher in the low-risk group, whereas the MES/CL subtype proportion was higher in the high-risk group in both male and female cohorts (Supplementary Figure S5F for males and Supplementary Figure S5G for females). MES and CL subtypes tended to have higher risk scores than the PN subtype in males (Supplementary Figure S5H) and females (Supplementary Figure S5I).

The expression of PN and MES marker genes indicated a clear association between risk score levels and PN/MES hallmarks, with MES marker genes enriched in high-risk-score GBMs and PN marker genes in the low-risk-score cohort (Figure 4N). To functionally validate the biological roles of the 10 risk-score-associated genes in the GBM PN/MES subtype modulation, we selected two representative genes from the risk score formula, FGFR3 (a positive variable) and COL6A3 (a negative variable), for in vitro characterization. Notably, the knockdown of FGFR3 in MES GBM cells (GSC267) significantly downregulated the MES marker CD44 while upregulating the PN marker SOX2. Conversely, COL6A3 knockdown in PN-type cells (GSC8-11) reduced SOX2 expression but enhanced CD44 levels, demonstrating bidirectional regulation of subtype-specific markers by these risk-score genes. Additionally, an evaluation of the tumor mutation burden (TMB) showed that the IDH1 mutation was among the top 20 most frequent mutations in low-score GBMs, which correlated with better survival for patients with a low-risk score (Supplementary Figure S5L,M). In general, these results suggest that the circadian risk score is a robust predictor of prognosis and molecular subtype in GBM patients.

### 2.4. Comparison of CCG Patterns, CRG Patterns, Circadian Risk Score, and Their Association with Fundamental Characteristics of GBM

**Figure 5:**
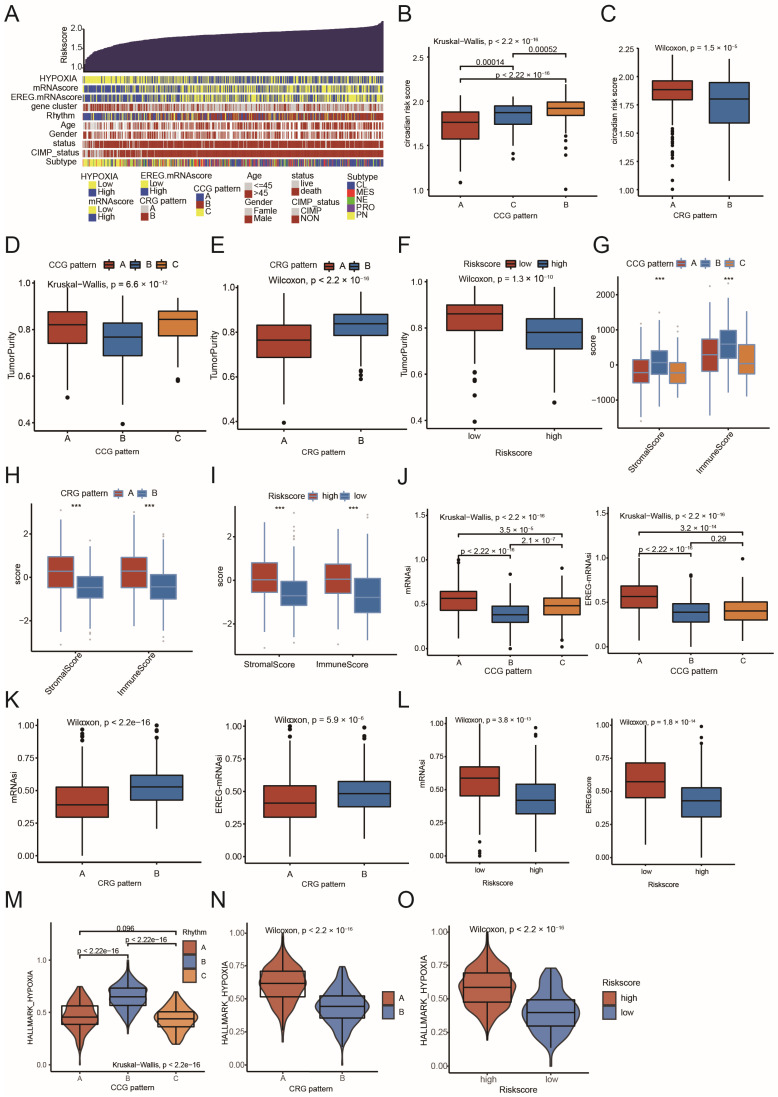
Comparison of CCG patterns, CRG patterns, circadian risk score, and their association with fundamental characteristics of GBM: (**A**) an overview of the association between circadian risk score and other patient annotations in MDSet cohort; (**B**) differences in circadian risk score among three circadian core-gene patterns (Kruskal–Wallis test); (**C**) differences in circadian risk score between two circadian-related gene patterns (Kruskal–Wallis test); (**D**) the relative tumor purity score of the three circadian core-gene patterns (Kruskal–Wallis test); (**E**) the tumor purity score of the two circadian-related gene patterns (Kruskal–Wallis test); (**F**) differences in tumor purity between circadian risk score high and low group (Kruskal–Wallis test); (**G**) the relative stromal score and immune score of the three circadian core-gene patterns calculated by ESTIMATE (Kruskal–Wallis test); (**H**) the relative stromal score and immune score of the two circadian-related gene patterns calculated by ESTIMATE (Kruskal–Wallis test); (**I**) differences in stromal score and immune score between circadian risk score high and low group (Kruskal–Wallis test); (**J**) the stemness indexes (mRNAsi and EREG-mRNAsi) of the three circadian core-gene patterns (Kruskal–Wallis test); (**K**) the stemness indexes (mRNAsi and EREG-mRNAsi) of the two circadian-related gene patterns (Kruskal–Wallis test); (**L**) the stemness indexes (mRNAsi and EREG-mRNAsi) between circadian risk score high and low group (Kruskal–Wallis test); (**M**) the hypoxia score of the three circadian core-gene patterns (Kruskal–Wallis test); (**N**) the hypoxia score of the two circadian-related gene patterns (Kruskal–Wallis test); (**O**) differences in hypoxia score between circadian risk score high and low group (Kruskal–Wallis test). *** *p* < 0.001.

The heatmap (Figure 5A) and alluvial diagram (Supplementary Figure S6A) were utilized to compare circadian core-gene patterns (CCG Patterns A, B, and C), circadian-related gene patterns (CRG Patterns A and B), and the circadian risk score. The cohorts with the worst prognosis, including CCG Pattern B, CRG Pattern A, and the high-risk score group, exhibited low tumor purity scores (Figure 5D–F) and enriched immune/stromal cell infiltration (Figure 5G–I), with significant presence of antigen-presenting cells (APCs) and macrophages (including MES-like macrophages, Supplementary Figure S6B–D). Consistent with previous findings [16], cohorts with a higher proportion of the PN subtype (CCG Pattern A, CRG Pattern B, and the low-risk score group) presented the highest levels of stemness indices such as mRNAsi and EREG-mRNAsi (Figure 5J–L). Additionally, the cohorts with the worst prognosis also exhibited a marked hypoxic microenvironment (Figure 5M–O). These results highlight the relevance of the three models we established and suggest that CCG patterns, CRG patterns, and the circadian risk scoring system can stratify GBM patients according to distinct fundamental characteristics, including stemness, immune infiltration, and hypoxia.

### 2.5. The Role of the Risk Score in the Prediction of Therapeutic Benefits

**Figure 6:**
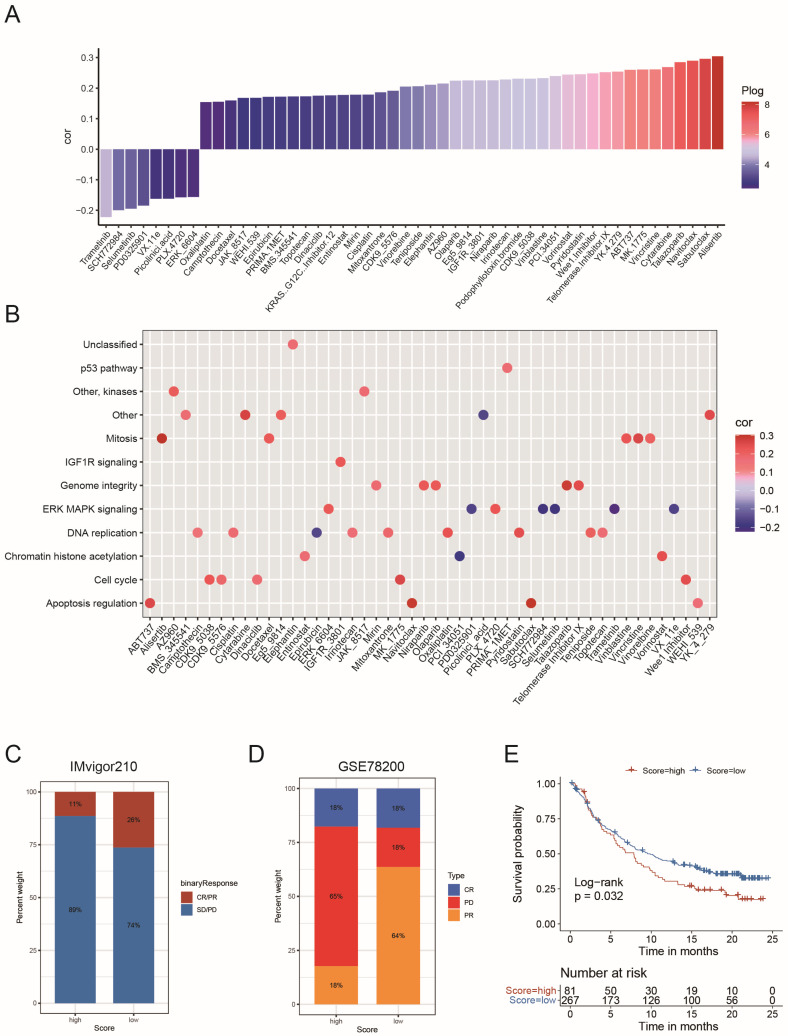
The relationship between circadian risk score and drug sensitivity and efficacy of immunotherapy: (**A**) The correlation between circadian risk score and drug sensitivity (AUC value) evaluated by the Spearman analysis. Each column represents a drug. The color of the column indicates the significance of the correlation. The height of the column indicates the correlation; (**B**) The pathways targeted by drugs in (**A**) were listed by dot plot. Drug names are listed on the horizontal axis and the signaling pathway targeted by the drug on the vertical axis; (**C**) Distribution of CR/PR and SD/PD cases in circadian risk score high and low group. Data were retrieved from anti-PD-L1 cohort (IMvigor210); (**D**) Distribution of CR, PD and PR cases in circadian risk score high and low group. Data were retrieved from anti-PD-1 cohort (GSE78200); (**E**) Survival analyses for circadian risk score high/low groups in the anti-PD-L1 cohort (IMvigor210, Log-Rank test, *p* = 0.032).

Spearman correlation analysis revealed that tumors with high-risk scores exhibited sensitivity to eight negatively correlated drugs, five of which targeted the MAPK signaling pathway (Figure 6A,B). Notably, MAPK signaling emerged as the most frequently targeted pathway for high-risk cancer-specific drugs, whereas low-risk tumors were primarily susceptible to drugs targeting DNA replication processes (Figure 6B). Given that 89% of MES GBM cases were classified as high-risk (Figure 4L), we further evaluated the efficacy of MAPK inhibitors in suppressing MES GBM viability. The well-characterized MAPK inhibitor trametinib significantly inhibited MES GBM cell proliferation but had minimal effects on PN GBM cells, suggesting its potential as a targeted therapy for high-risk patients (Supplementary Figure S6E). Evaluation of immune checkpoints in GBM revealed that most checkpoint molecules exhibited preferential expression in specific circadian core-gene patterns, circadian-related gene patterns, and risk score groups. This indicates that these GBM-stratifying models may aid in identifying targets for checkpoint blockade immunotherapy (Supplementary Figure S6F–H).

The radar chart showed that the risk scoring scheme correlated with the burden of neoepitopes, such as microsatellite instability (MSI) and tumor mutational burden (TMB), in several types of cancers, which robustly reflects the response to checkpoint blockade immunotherapy (Supplementary Figure S7A,B). Additionally, 22 of 32 cancers showed a significant correlation between the risk score and CD274 expression (Supplementary Figure S7C). In a pan-cancer study, the risk score was significantly associated with immune cell infiltration and cell stemness in most cancer types (Supplementary Figure S7D,E), suggesting that the scoring scheme could potentially reflect the response to checkpoint blockade immunotherapy. In both the anti-PD-L1 cohort (IMvigor210) and the anti-PD-1 cohort (GSE78220), patients with low circadian risk scores exhibited marked clinical benefits from immune therapy (Figure 6C,D). Survival data suggested that a lower risk score was a favorable prognostic hallmark for patients receiving anti-PD-L1 therapy (*p* = 0.032, Figure 6E). Although higher risk scores indicated shorter survival in the anti-PD-1 cohort, the survival differences between high- and low-risk score groups did not reach statistical significance (*p* = 0.099, Supplementary Figure S7F). In summary, the circadian risk score is a valuable predictor of chemo- and immune therapy effectiveness for individual GBM patients.

## 3. Discussion

In this study, we identified and analyzed the expression profiles of 17 circadian core-genes and 32 circadian-related genes in GBM. Two subtyping systems were established named circadian core-gene pattern and circadian-related gene pattern, respectively. GBM patients with distinct molecular profiles, malignant characteristics and prognoses could be classified by these patterns. We also developed a circadian risk scoring system, which enables the calculation of an individual patient’s circadian risk score based on the expression of merely 10 circadian-related genes. GBM patients with lower circadian risk scores were associated with prolonged overall survival and less aggressive molecular profiles, and vice versa. Further analysis verified that GBM patients who present high risk scores probably benefit from MAPK-targeted drugs, whereas patients with low risk scores are supposed to respond better to anti-PD-1/L1 immunotherapy.

The past decades have witnessed considerable advances in the classification of GBM. With the rapid improvement in next-generation sequencing, there is a growing trend toward achieving unprecedented accuracy and simplicity in classification, often using minimal gene sets [3]. For example, Du et al. reported a classification system for GBM based on 13 m6A RNA methylation regulators [17], while Sun et al. established a classification system based on 27 anoikis-related genes [18]. Our study adds to this body of work by demonstrating that a conservative set of circadian rhythm genes—including core genes (e.g., CLOCK, ARNTL, CRYs) and circadian-related genes (filtered from DEGs among three circadian core-gene patterns)—can effectively classify GBM patients. Among three circadian core-gene patterns (CCG Patterns A, B and C) or two circadian-related gene patterns (CRG Patterns A and B), different predominant molecular subtypes and distinct overall survival were shown. We also organized a classification system according to the score valued by merely 10 circadian-related genes, presenting efficiency for prognosis prediction and PN subtype evaluation. In addition, this scoring scheme could quantify every single GBM sample, which makes it possible to use a collection of 10 genes for accurate evaluation of molecular profiles and prognosis in individual GBM patients.

Emerging evidence has demonstrated significant sexual dimorphism in circadian regulation, with distinct impacts on tumor biology and drug metabolism [19]. Notably, studies have identified sex-dependent circadian fluctuations in P-glycoprotein expression that correlate with drug resistance [20]. Furthermore, the bidirectional relationship between circadian clocks and sex hormone regulation has been shown to influence tumor progression [21,22]. In the present study, we systematically evaluated potential sex differences in our circadian classification system. Initial analyses revealed no significant gender-based variations in circadian core-gene pattern distribution. Subsequent sex-stratified validation demonstrated consistent prognostic performance across both male and female cohorts, with Pattern B maintaining its association with worse outcomes in all subgroups. Similarly, the risk-scoring system effectively stratified survival outcomes in both sexes. These findings suggest that our models are robust across genders, though further investigation into sex-specific circadian mechanisms in GBM is warranted.

GBM has long been characterized by robust circadian rhythms. Many circadian clock genes were oncogenes or tumor suppressors in GBM. Accordingly, our study recognized CLOCK and BMAL1 as unfavorable prognostic factors for GBM, while numerous genes in the feedback loops such as CRY1, NRID2 and RORC [23] were correlated with better prognosis. It seems reasonable to target circadian rhythm for GBM therapy. Actually, numerous efforts have been made to modulate the circadian clock by directly targeting circadian clock genes. Recent preclinical work by Sulli et al. [14] showed that pharmacological activation of REV-ERB can inhibit GBM growth. Other studies suggested depletion of core genes CLOCK and BMAL1 impairing tumor progression [7]. However, given the complexity of circadian gene networks and the heterogeneity of the endogenous clock for individuals, targeting single genes in this network might not alter the tumors’ intrinsic biological clock system. Therefore, it is imperative to stratify GBM patients based on the expression pattern of circadian gene networks instead of the expression level of one single circadian gene. In our study, a set of 10 circadian-related genes were filtered in the differential expression genes among three distinct subgroups classified by the expression pattern of circadian gene networks. These genes were core DEGs between three circadian core-gene patterns, which could be recognized as parameters comprehensively reflecting the overall expression profile of the circadian gene network. Actually, targeting two genes in this scoring system could induce molecular subtype transition in GBM cells, which suggested a global regulation of GBM molecular profiles by core DEGs among circadian core patterns. These findings provided new insight into circadian-targeting therapy for GBM.

In addition to directly targeting circadian-related genes, our scoring system provided an opportunity for targeted therapy selection based on circadian risk score. For example, after quantification of 10 genes and calculation of the risk score, patients who present high-risk scores would benefit from MAPK-targeted drugs whereas a patient with a low-risk score is supposed to respond better to anti-PD-1/L1 immunotherapy, partially due to high expression of the PDCD1 gene in this group of patients. Particularly noteworthy was the finding that 89% of MES-subtype GBMs fell into the high-risk category, suggesting our score could help guide treatment selection for this particularly aggressive subtype. This model would enlarge the potential drug pool selected for individual GBM patients.

Chronomedicine is a recently established subject that focuses on determining whether a patient would benefit from a time-of-day-based treatment regimen. The efficacy of chemo- and radiotherapy for cancer fundamentally depends on their capacity to induce DNA damage. Given the circadian clock’s well-documented role in regulating both cell cycle progression and DNA damage response pathways, there exists a compelling biological rationale for timing chemoradiotherapy for GBM, especially for those subtypes resistant to chemoradiation (MES GBM) [24]. However, it remained controversial whether GBM patients benefit from time-of-day treatment [25]. Clinical trials on this topic mainly focused on timed delivery of medications in accordance with the clock time [26,27]. Accumulating evidence suggests that, in addition to the external clock time that we follow in daily life, endogenous clocks for one patient might be a more important factor determining sensitivity to chrono-chemoradiotherapy, which researchers called individual chronotype [24]. Our study provided a novel way to recognize the chronotype for GBM, namely circadian core gene (CCG) patterns. For example, GBM in CCG Patterns B and C showed higher Arntl gene expression than GBM in Pattern A. Previous preclinical data from Slat et al. demonstrated that the sensitivity of GBM cell to temozolomide (TMZ) peaked during the circadian phase corresponding to maximal Arntl expression [28]. Therefore, we supposed that GBM patients who displayed a chronotype similar to CCG Patterns B and C would express more Arntl and benefit from temozolomide chemotherapy. In addition, this group of patients might better respond to chrono-temozolomide treatment when Arntl is at its peak transcript level, probably from 3 p.m. to 9 p.m. according to previous studies [29,30]. Future clinical trials could utilize our circadian core gene patterns to stratify patients for chronotherapy. This approach may overcome therapeutic resistance by targeting biologically vulnerable subgroups, thereby translating circadian-driven susceptibility into measurable survival benefits.

Several intratumoral characteristics, such as stemness, hypoxia and immune infiltration could reflect tumor malignancy and are closely related to the molecular profiles of GBM [31]. Single-cell sequencing is the optimum strategy to evaluate these characteristics within the GBM. Indeed, we analyzed single-cell sequencing data and confirmed specific associations of these characteristics in GBM cells with robust circadian rhythm signatures. However, clinically leveraging single-cell sequencing to evaluate these characteristics in individual GBM seems to be time-consuming and expensive. Moreover, a large proportion of GBM atlas projects launched before were based on bulk sequencing data that could not be analyzed on a single-cell scale. Since we have found that GBM patients with higher circadian risk scores would present severe hypoxia, high immune infiltration (especially for MES-like macrophages), low tumor purity and low stemness, we suppose that our ten-gene-based scoring system might have the potential to evaluate these intratumor features when expression of ten genes was provided.

## 4. Methods

### 4.1. Data Collection and Processing

**Figure 7:**
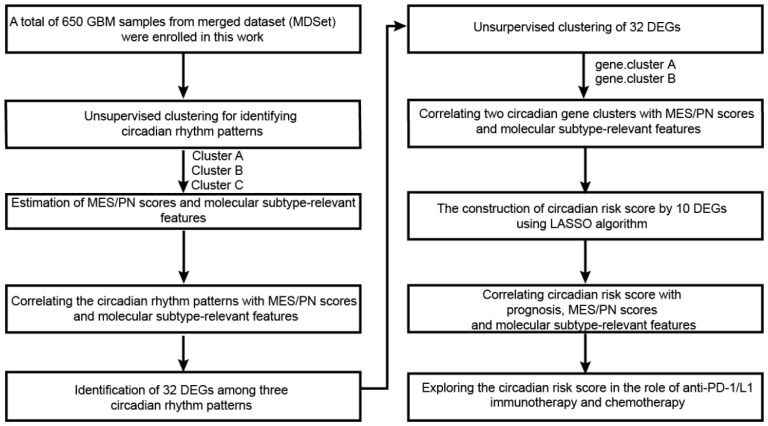
The workflow chart for this study.

The study workflow chart is shown in Figure 7. Public gene-expression data and full clinical annotation were searched in TCGA (the Cancer Genome Atlas) GBM database and CGGA (Chinese Glioma Genome Atlas) GBM database. TCGA is the largest global database for solid tumors, while CGGA is the most authoritative dataset for glioma in China [32]. These databases are widely used in GBM genomic and transcriptomic analysis [33]. All GBM samples with full survival and subtype information in TCGA-GBM (n = 169) and CGGA-GBM (n = 237) databases were included for analysis. The cohort and demographic information were available from these databases.

Referring to previous research [18,34,35], datasets published by others’ studies were gathered for analysis in order to verify the findings in larger cohorts. In total, microarray data for GBM samples from 6 independent pieces of research were merged as a whole cohort, named merged dataset (MDSet, n = 650). MDSet merged the GBM sample in the following dataset including Rembrandt (n = 219) [36], Gravendeel (n = 159) [37], Freije (n = 59) [38], Murat (n = 80) [39], Joo (n = 57) [40] and Phillips (n = 76) [41], all of which were downloaded from GlioVis (http://gliovis.bioinfo.cnio.es/ (accessed on 1 December 2024)). All samples from these 6 datasets include clinical annotation such as survival and molecular subtype information. The somatic mutation counts and copy number variation (CNV) were obtained from TCGA database. We used sva Package’s “ComBat” algorithm to correct nonbiotechnology deviations causing batch effects. R Bioconductor packages and R (version 4.10) were used to analyze the data.

### 4.2. Unsupervised Clustering for 17 Circadian Rhythm Genes

Circadian rhythm genes were extracted from previous study [15]. In total, 17 genes with expression information in six datasets in MDSet were utilized for further analysis. The expression matrix of these 17 genes was extracted from 650 samples in MDset. R4.1.2 software was used for unsupervised clustering. The R package ConsensusClusterplus was applied to cluster analysis of 17 genes in 650 GBM samples. This R package functions for determining cluster number and class membership by stability evidence. We applied the ConsensusClusterPlus package to execute the steps above with one thousand repetitions to guarantee classification stability.

### 4.3. Single-Cell RNA Sequencing Analysis

The single-cell RNA-sequencing of gliomas was downloaded from Gene Expression Omnibus (GEO, https://www.ncbi.nlm.nih.gov/geo/ (accessed on 1 December 2024), GSE138794) and analyzed using R package “Seurat 4.1.0”. A gene signature was established based on 17 circadian rhythm genes and used for cell annotation in single-cell sequencing. Method “UMAP” was applied for the visualization of different cell clusters. Method “tSNE” was applied for the visualization of different cell clusters. R package “irGSEA” was used to calculate and visualize the enrichment scores of the Verhaak_GBM_MES signature, hypoxia signature and stemness signature by method “UCell”.

### 4.4. Gene Functional Annotation Based on Gene Set Variation Analysis (GSVA)

To analyze differences in biological processes between circadian core-gene patterns, GSVA enrichment analysis was performed via the “GSVA” R package. We downloaded the geneset “c5.go.bp.v7.4” as well as “c2.cp.kegg.v7.4” from the Molecular Signatures Database (MSigDB, https://www.gsea-msigdb.org/gsea/msigdb/index.jsp (accessed on 1 December 2024)) for GSVA analysis.

### 4.5. TME Cell Infiltration and Hypoxia Estimation

The 28 immune-related cells and immune-related gene signatures were obtained from the dataset of Bindea et al. [42]. First, single-sample gene set enrichment analysis (ssGSEA) was used to identify tumor immune infiltrating cell abundance in GBM samples based on the above 28 signatures. Next, Estimation of Stromal and Immune Cells in Malignant Tumor Tissues Using Expression Data (ESTIMATE) algorithm was used to infer the degree of infiltration of stroma or immune cells into GBM based on RNA sequence profiles [43]. Using ESTIMATE package, we calculated the stromal and immune score for each sample. Then, we investigated the relationship between each GBM cluster and Stromal/Immune score generated by ESTIMATE. HALLMARK_HYPOXIA.v7.5.1 gene set (http://www.gsea-msigdb.org, accessed on 1 December 2024) was used for annotation of hypoxia among the GBM samples. ssGSEA was performed as above-mentioned.

### 4.6. Identification of DEGs Among Circadian Core-Gene Patterns

To identify circadian-related genes, DEGs among 3 different circadian core-gene patterns were arranged via the limma R package. The significance criterion for DEGs was *p* value < 0.05.

### 4.7. Constructing the Circadian Risk Scoring System to Evaluate Individual GBM Samples

The circadian risk scoring system was established as previously reported [34,35,44,45]. Briefly, the ‘sample’ function in R was used to randomly divide the total of 650 GBM samples in MDSet into two parts (7:3) for training and testing. TCGA and CGGA samples were used as external test sets for scoring system. A total of 32 DEGs among 3 different circadian core-gene patterns were arranged. The prognostic analysis for each gene was performed using the least absolute shrinkage and selection operator (LASSO) on the COX regression model, which was calculated by R package glmnet. The ten genes with the significant prognosis were extracted for further analysis and the regression coefficient for each gene was also calculated by LASSO algorithm. We therefore deduced the following formula:

circadian risk score = CDH18 × (−0.002385586) + CENPF × (0.037396591)+ CHI3L1 × (0.068107208) + COL6A3 × (−0.012696497) + FGFR3 × (0.041623413) + GPR37 × (0.005960511) + IL13RA2 × (0.027679954) + MYBPC1 × (0.033495716) + RALYL × (−0.005094129) + SOX11 × (0.011377282)

The expression of 10 genes is the variable and regression coefficient for each gene calculated by LASSO algorithm was set as weighting coefficients. Since CHI3L1 is a well-characterized marker for MES subtype, the other two variables with the largest absolute regression coefficients in the formula, FGFR3 and COL6A3, were selected for further in vitro study.

### 4.8. Analyzing the Association Between Circadian Risk Score and Response to Immunotherapy

Two independent immunotherapy cohorts, advanced urothelial tumor with atezolizumab treatment (IMvigor210 cohort, http://research-pub.gene.com/IMvigor210CoreBiologies/packageVersions, accessed on 1 December 2024) and metastatic melanoma with pembrolizumab treatment (GSE78220 cohort), were obtained. The samples’ circadian risk score was calculated and the response to immunotherapy was compared between high and low risk score clusters.

### 4.9. Association Analysis Between Drug Sensitivity and Circadian Risk Score

Cancer Cell Line Encyclopedia (CCLE) RNA-seq data were obtained from https://portals.broadinstitute.org/ccle/ (accessed on 1 December 2024). The AUC value, which indicated cellular sensitivity to anti-tumor drugs, was obtained from Genomics of Drug Sensitivity in Cancer (GDSC; https://www.cancerrxgene.org/downloads, accessed on 1 December 2024). The data recording drug targeting pathways were also obtained from Genomics of Drug Sensitivity in Cancer (GDSC; https://www.cancerrxgene.org/downloads, accessed on 1 December 2024). Spearman correlation analysis was applied to estimate the association between drug sensitivity and the circadian risk score. A positive correlation indicates that cell lines with high circadian risk scores have higher AUC values and lower sensitivity to specific drugs, whereas a negative correlation indicates that cell lines with high circadian risk scores have lower AUC values and higher sensitivity to specific drugs.

### 4.10. Nomogram Construction

R package “rms” was utilized to build the nomogram adopting variables with predictive significance in multivariate analysis (*p* < 0.05). Calibration curves were used to assess the consistency between predicted and actual survival outcomes. Furthermore, time-dependent ROC curves were applied to compare the predictive accuracy of nomograms.

### 4.11. Western Blot Analysis

Cells were washed with cold PBS and lysed with RIPA buffer containing 1% protease and phosphate inhibitor cocktail (P8340; Sigma–Aldrich, St. Louis, MO, USA). After sodium dodecyl sulfate-polyacrylamide gel electrophoresis (SDS–PAGE), proteins were transferred to polyvinylidene fluoride (PVDF) membranes. Then, the membranes were cut into strips and incubated with primary antibodies at 4 °C overnight and were then incubated with secondary antibodies. We examined protein expression using an Odyssey fluorescence scanner (ChemiDoc XRSþ, Bio-Rad, Hercules, CA, USA). Primary antibodies: Rabbit anti-FGFR3 (ab133644), Rabbit anti-COL6A3 (ab275680), Rabbit anti-CD44 (ab243894), Rabbit anti-SOX2 (ab92494), Mouse anti-β-actin (ab6276).

### 4.12. Ethynyl-2′-Deoxyuridine (EdU) Cell Proliferation Assay and Trametinib Pretreatment

EdU assay kit (Ribobio, Guangzhou, China) was used to test the cell proliferation ability according to the manufacturer’s instructions. Trametinib were purchased from MedChemExpress (MedChemExpress, Monmouth Junction, NJ, USA). Dimethyl sulfoxide was used to dissolve trametinib powder to an initial concentration of 10 mM and preserved in −80 °C. For GBM cells pretreatment, GBM cells were seeded into wells of poly-l-ornithine precoated 12-well plates. The amount of 50 nM trametinib was used for pretreatment for 72 h. Cells were then incubated with 200 μL of 5-ethynyl-20-deoxyuridine for 2 h at 37 °C. Nuclei were counterstained with Hoechst 33342. Representative images were obtained with a Leica inverted fluorescence microscope (Leica, Barrington, IL, USA).

### 4.13. Cell Transfection

For short-term knockdown of GBM cells, cells were transfected with siRNAs using the Lipofectamine 3000 kit (Invitrogen, Carlsbad, CA, USA) according to the manufacturer’s instructions. After 24 h transfection, the knockdown efficiency of FGFR3 and COL6A3 can be observed for further detection. Si-FGFR3-1: 5′-GCAUUGGAGGCAUCAAGCUTT-3′, si-FGFR3-2: 5′-GCUGAAAGACGAUGCCACUTT-3′, si-FGFR3-3: 5′-GCACACACGACCUGUACAUTT-3′. Si- COL6A3-1: 5′-CCGGAAGUGUCAAUUUCGCAGUCAU-3′, si-COL6A3-2: 5′-GGCUGGAAAUCGGACAGGAUCUUAU-3′, si-COL6A3-3: 5′-GCGACUUUGUAAUGAACCUAGUUAA-3′. NC: 5′-UUCUCCGAACGUGUCACGUTT-3′.

### 4.14. Statistical Analysis

The statistical analyses were generated by R-4.0.1. Distance and Spearman correlation analyses were applied to calculate 17 circadian rhythm gene expression correlation coefficients. Kruskal–Wallis as well as one-way ANOVA tests were applied to appraise difference comparisons of more than three collections. To assess the relationship between patient survival and the circadian risk score, the optimal cutoff point for survival information for each dataset was determined using the survminer R package. The log-rank test and Kaplan–Meier analysis were applied to assess survival among different patterns as well as circadian risk score groups. The hazard ratio (HR) of genes was calculated via a univariate Cox regression model. To evaluate whether the circadian risk score serves as an independent predictor for survival, sex, age, as well as IDH status were considered variables in the multivariate Cox regression model analysis. Statistical analysis was 2-sided, as well as *p* < 0.05 was regarded as statistically significant. The receiver operating characteristic (ROC) curve was used to evaluate the diagnostic value of circadian risk score, and the area under the curve (AUC) was quantified using the pROC R package.

## 5. Conclusions

In conclusion, the current study demonstrated the key role of circadian rhythm genes in the regulation of GBM molecular profiles. The circadian rhythm gene network could be a potential target for GBM therapy. It is noteworthy that the expression level of circadian core genes rhythmically fluctuated under the control of the biological clock. Therefore, temporal targeting of the circadian gene network should be considered. Controlling the timing of medication and treatment and capturing the optimal dosing window deserves deeper study in the future. Moreover, the newly established two patterns and circadian risk scores in this study provided opportunities for the evaluation of molecular profiles and therapy responsiveness in GBM and brought up a model for prognosis prediction and treatment outcome estimation for individual patients.

## Data Availability

The datasets generated and/or analyzed during the current study are available in the TCGA and GEO repository (https://www.cancer.gov/tcga; https://www.ncbi.nlm.nih.gov/geo/, accessed on 1 December 2024).

## Supplementary Materials

The following supporting information can be downloaded at: https://www.mdpi.com/article/10.3390/ijms26125873/s1.

## Abbreviations

APCs	Antigen-presenting cells
AUCs	area under the curves
CCLE	Cancer Cell Line Encyclopedia
CGGA	Chinese Glioma Genome Atlas
CL	Classical
CNS	Central Nervous System
CNV	copy number variation
DEGs	differential expressing genes
GBM	glioblastoma
GDSC	Genomics of Drug Sensitivity in Cancer
GEO	Gene Expression Omnibus
GSVA	gene set variation analysis
HR	hazard ratio
IDH	Isocitrate dehydrogenase
KEGG	Kyoto Encyclopedia of Genes and Genomes
LASSO	Least Absolute Shrinkage and Selection Operator regression
MES	Mesenchymal
MDSet	merged dataset
PCA	principal component analysis
PN	Proneural
ROC	receiver operating characteristic
ssGSEA	single-sample gene set enrichment analysis
TCGA	The Cancer Genome Atlas
