# Supplementary material for: The Circadian Rhythm Gene Network Could Distinguish Molecular Profile and Prognosis for Glioblastoma
Source: Int J Mol Sci. 2025 Jun 19;26(12):5873. doi: 10.3390/ijms26125873 (PMC12193191; doi:10.3390/ijms26125873)
Supplement: Supplementary file 1 [file ijms-26-05873-s001.zip › ijms-3503648-supplementary.pdf]

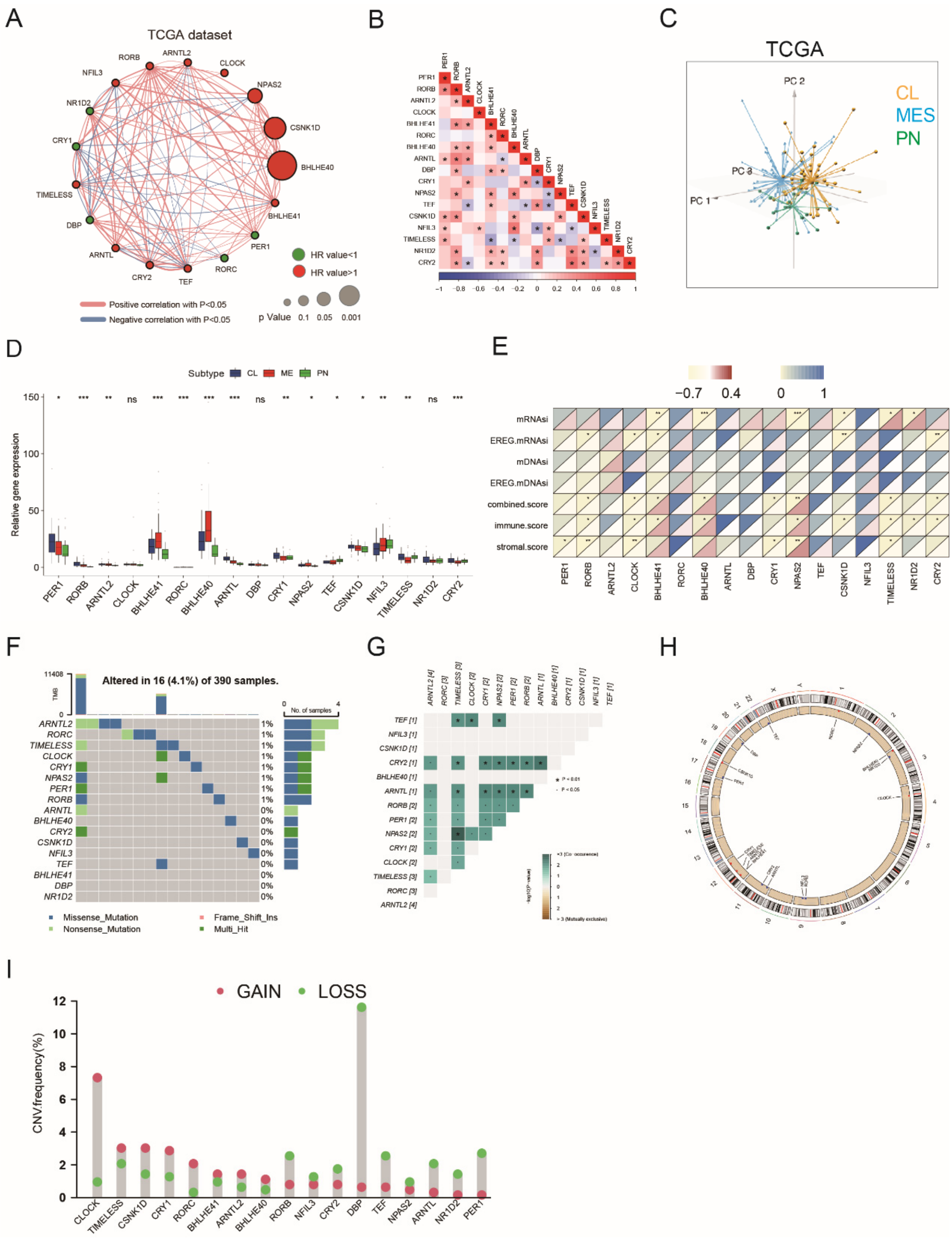

Supplementary Figure S1. Landscape of genetic and expression variation of circadian rhythm genes in GBM.

A. The interaction between circadian rhythm genes in GBM cases in TCGA cohort. The circadian rhythm genes with HR value <1 and >1 was depicted by circles in green and red, respectively. The lines connecting circadian rhythm genes represented their

interaction with each other. The size of each circle represented the p value for HR value.

B. Spearman correlation analysis for 17 circadian rhythm genes. Data were retrieved from TCGA.

C. Principal component analysis (PCA) for the expression profiles of 17 circadian rhythm genes to distinguish CL, PN, MES GBM in TCGA cohort. Three subgroups without intersection were identified, indicating the CL, PN, MES GBM were well distinguished based on the expression profiles of circadian rhythm genes. CL, PN, MES GBM were marked with yellow, blue and green, respectively.

D. The relative gene expression of 17 circadian rhythm genes in CL, PN, MES GBM (Kruskal-Wallis test, $*p < 0.05$; $**p < 0.01$; $***p < 0.001$). Data were retrieved from TCGA cohort.

E. Correlation between 17 circadian rhythm genes and stemness indices in TCGA cohort. The upper part of each grid showed the p value, and the bottom part showed the correlation coefficient. The asterisks represented the statistical p value. (Pearson test, $*p < 0.05$; $**p < 0.01$; $***p < 0.001$)

F. The mutation frequency of 17 circadian rhythm genes in samples from the TCGA cohort. Each column represented an individual patient. The upper bar plot indicated mutation-accumulation. The bar plot on the right indicated the proportion of each variant type with the number above representing mutation frequency.

G. Co-occurrence and mutually exclusive of mutation among 14 circadian rhythm genes with mutation. Data were evaluated by Spearman analysis. Data were retrieved

from TCGA.

H. The location of CNV alteration of circadian rhythm genes on 23 chromosomes. Data were retrieved from TCGA.

I. The CNV frequency of circadian rhythm genes in GBM patients. The height of each column represented the alteration frequency. The amplification frequency, red dot; The deletion frequency, green dot. Data were retrieved from TCGA.

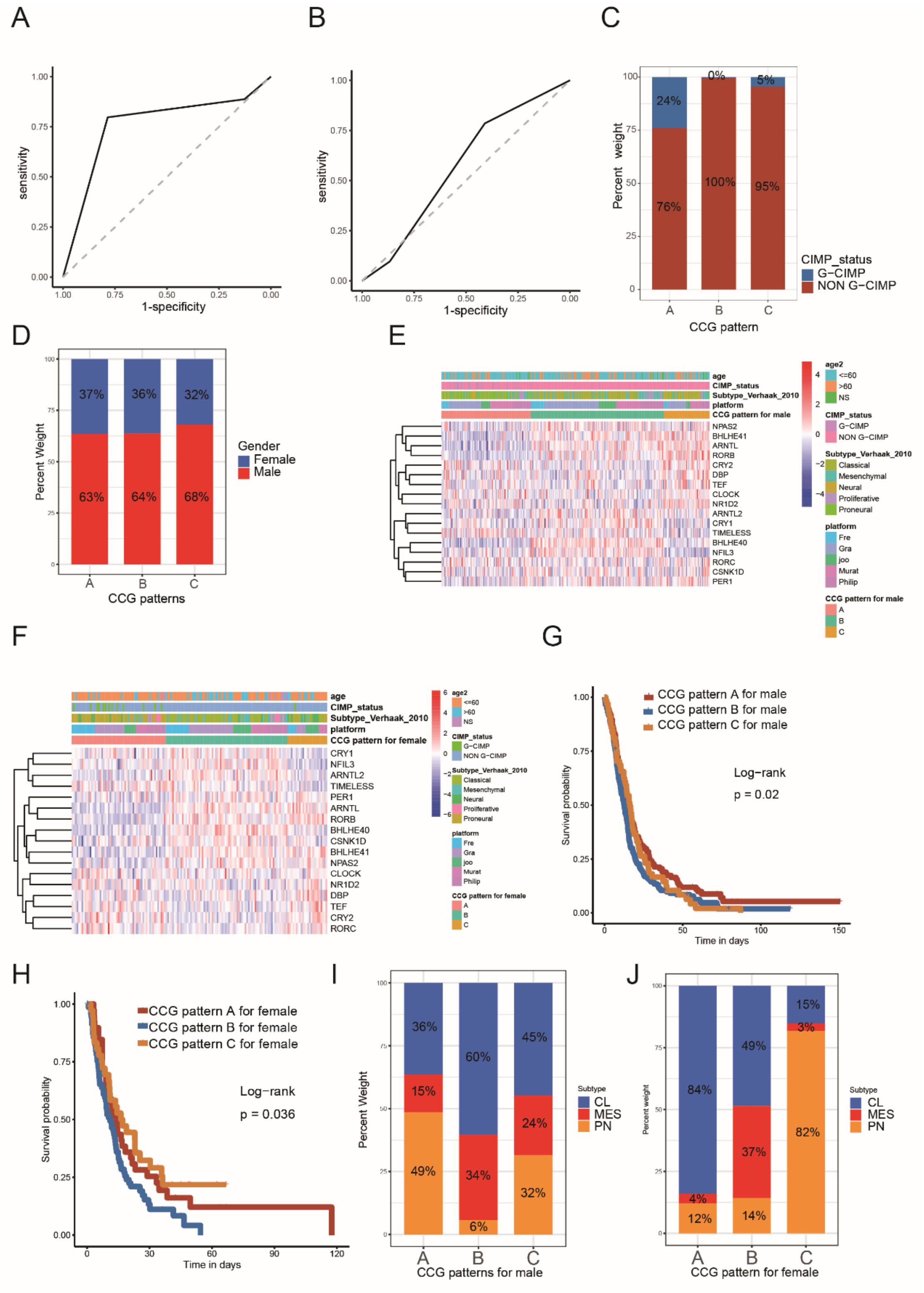

Supplementary Figure S2. Circadian core-gene patterns and pathological features of each pattern.

A. Receiver operating characteristic (ROC) curve for PN subtype prediction by

circadian core-gene patterns in MDSet dataset (AUC=0.76).

B. ROC curve for MES subtype prediction in MDSet dataset (AUC=0.57).

C. The proportion of G-CIMP and non-G-CIMP cases in the three circadian core-gene patterns.

D. Proportion of male and female patients in each CCG patterns.

E. Unsupervised clustering of 17 circadian rhythm genes in male patients MDSet cohort.

F. Unsupervised clustering of 17 circadian rhythm genes in female patients MDSet cohort.

G. Survival analyses for circadian core patterns A, B and C for male in the MDSet-male cohort (Log-Rank test, $p = 0.02$).

H. Survival analyses for circadian core patterns A, B and C for female in the MDSet-female cohort (Log-Rank test, $p = 0.036$).

I. Percentage of CL, MES, PN subtype in CCG pattern A, B and C for male in the MDSet-male cohort.

J. Percentage of CL, MES, PN subtype in CCG pattern A, B and C for female in the MDSet-female cohort.

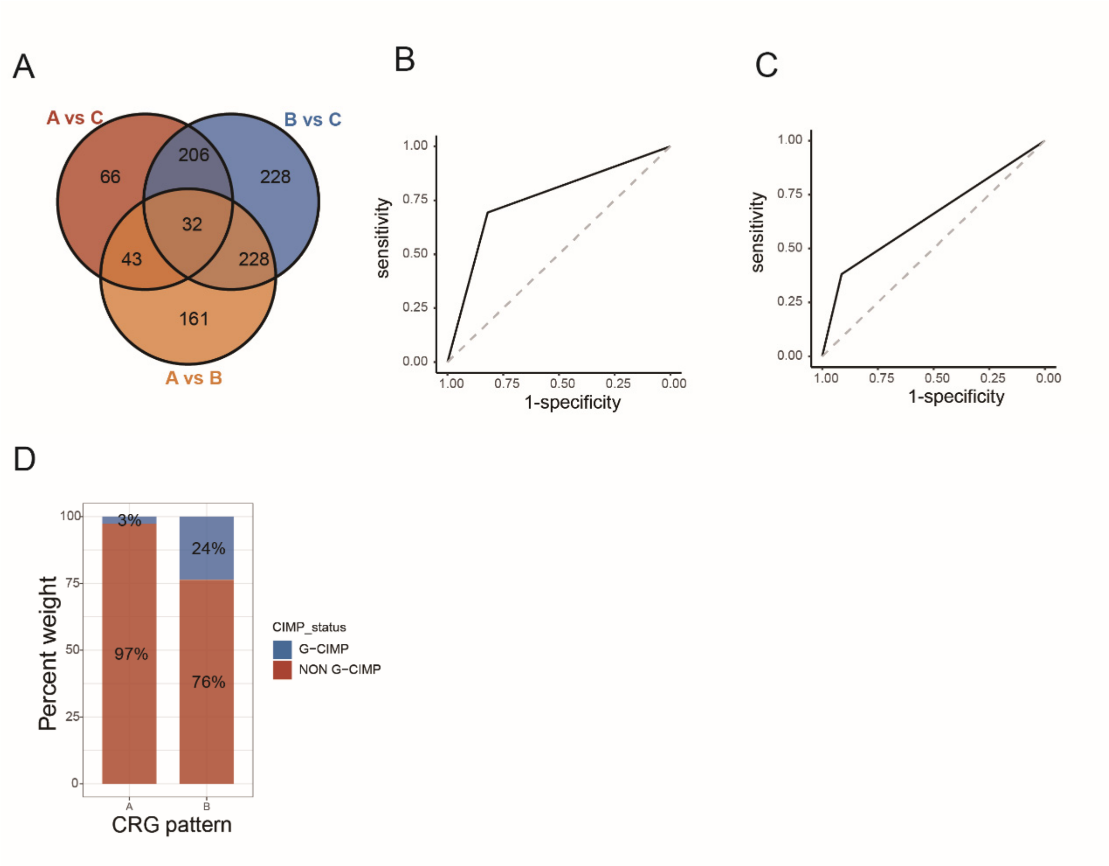

Supplementary Figure S3. Circadian-related gene patterns and pathological features of each pattern.

A. Venn diagram depicted overlapped DEGs among circadian core-gene patterns A, B and C.

B. ROC curve for PN subtype prediction by circadian-related gene patterns in MDSet dataset (AUC=0.76).

C. ROC curve for MES subtype prediction in MDSet dataset (AUC=0.65).

D. Distribution of G-CIMP and non-G-CIMP GBM in the two circadian-related gene patterns.

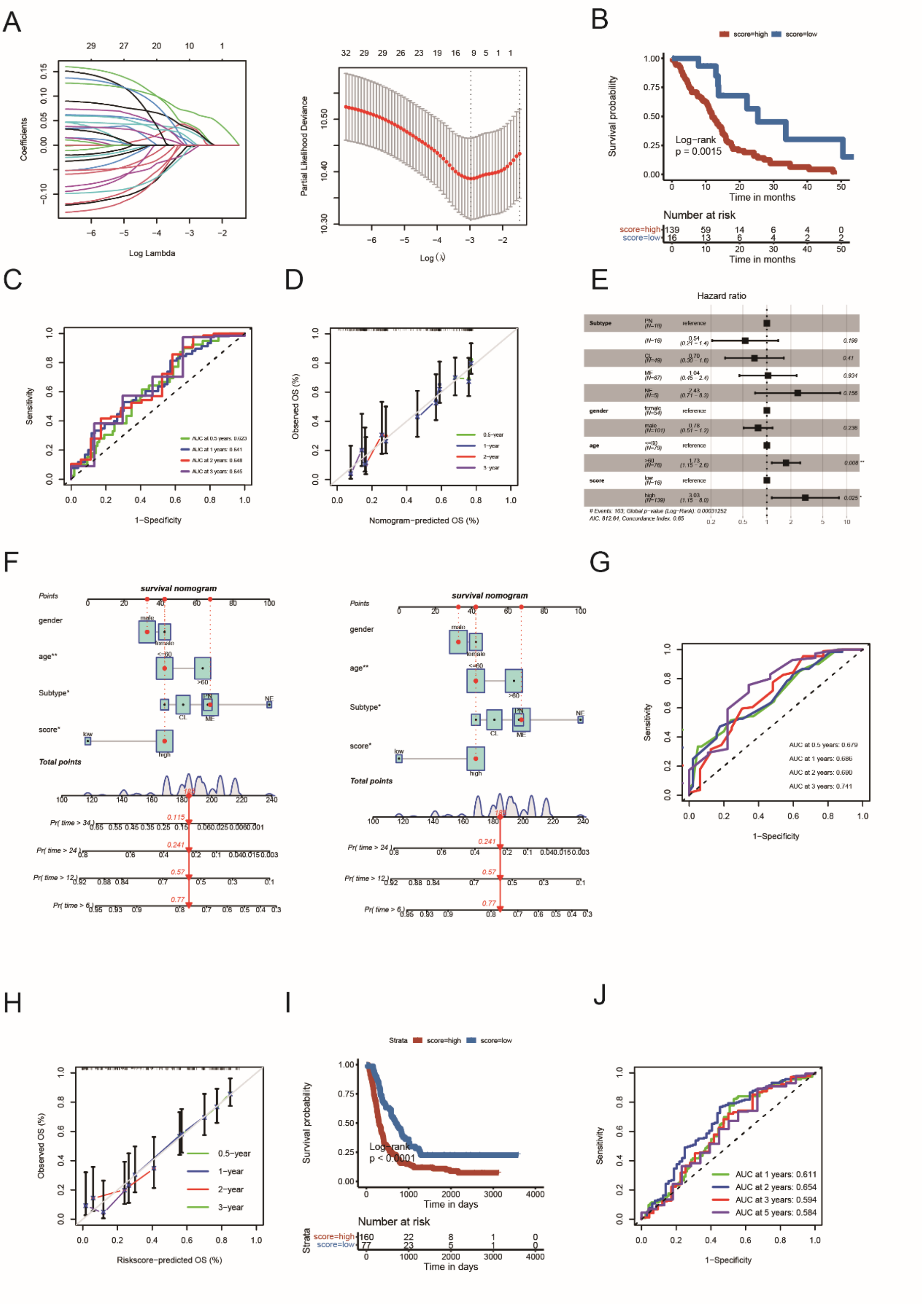
A
Coefficients
Log Lambda
Partial Likelihood Deviance
Log (λ)
B
score=high
score=low
Survival probability
Log−rank
p = 0.0015
Time in months
Number at risk
score=high 139 59 14 6 4 0
score=low 16 13 6 4 2 2
C
Sensitivity
1−Specificity
D
Observed OS (%)
Nomogram−predicted OS (%)
E
Hazard ratio
F
survival nomogram
Points
gender
age**
Subtype*
score*
Total points
G
Sensitivity
1−Specificity
AUC at 0.5 years: 0.679
AUC at 1 years: 0.686
AUC at 2 years: 0.690
AUC at 3 years: 0.741
H
Observed OS (%)
Riskscore−predicted OS (%)
0.5−year
1−year
2−year
3−year
I
Strata
score=high
score=low
Survival probability
Log−rank
p < 0.0001
Time in days
Number at risk
score=high 160 22 8 1 0
score=low 77 23 5 1 0
J
Sensitivity
1−Specificity
AUC at 1 years: 0.611
AUC at 2 years: 0.654
AUC at 3 years: 0.594
AUC at 5 years: 0.584

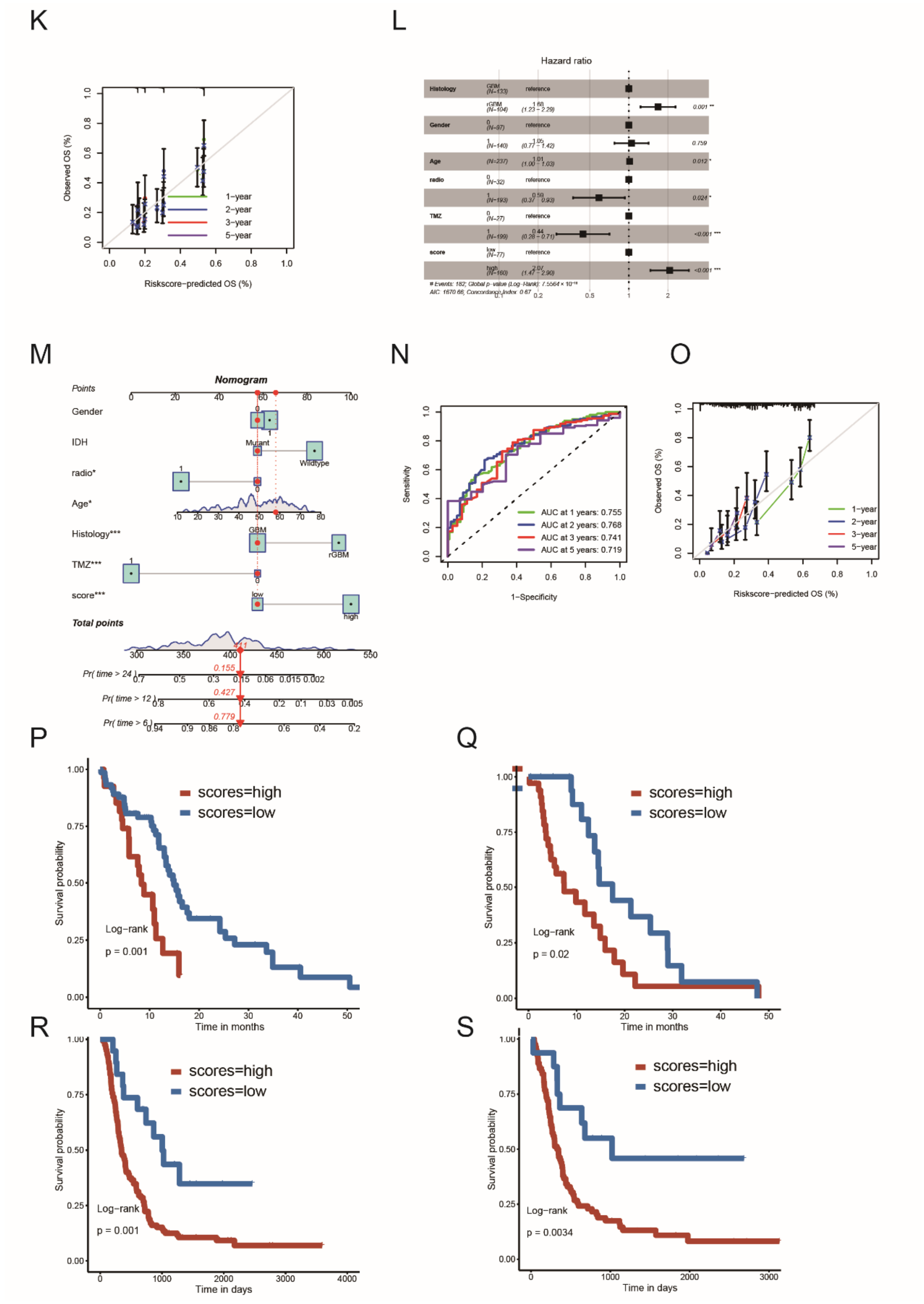

Supplementary Figure S4. Construction of circadian risk score and evaluation of its predictive potentiality.

A. LASSO machine learning algorithms were used for feature selection.

B. Survival analyses for circadian risk score high/low groups in the TCGA cohort (Log-Rank test, $p = 0.0015$).

C. ROC curve for 0.5, 1, 2, 3-year survival prediction in test set from TCGA cohort. The accuracy was equal to AUC.

D. Calibration curves for 0.5, 1, 2, 3-year survival prediction in test set from TCGA cohort.

E. Multivariate Cox regression model analysis of clinicopathological characteristics and circadian risk score with overall survival in the TCGA cohort.

F. Nomograms for predicting the probability of patient mortality in TCGA cohort based on circadian risk score, gender, subtype and age.

G. ROC curve for 0.5, 1, 2, 3-year survival prediction by nomogram-related score in TCGA cohort. The accuracy was equal to AUC.

H. Calibration curves for 0.5, 1, 2, 3-year survival prediction by nomogram-related score in TCGA cohort.

I. Survival analyses for circadian risk score high/low groups in the CGGA cohort (Log-Rank test, $p < 0.0001$).

J. ROC curve for 1, 2, 3, 5-year survival prediction in test set from CGGA cohort. The accuracy was equal to AUC.

K. Calibration curves for 1, 2, 3, 5-year survival prediction in test set from CGGA cohort.

L. Multivariate Cox regression model analysis of clinicopathological characteristics and circadian risk score with overall survival in the CGGA cohort.

M. Nomograms for predicting the probability of patient mortality in CGGA cohort based on circadian risk score and other clinicopathological features.

N. ROC curve for 1, 2, 3, 5-year survival prediction by nomogram-related score in CGGA cohort. The accuracy was equal to AUC.

O. Calibration curves for 1, 2, 3, 5-year survival prediction by nomogram-related score in CGGA cohort.

P. Survival analyses for circadian risk score high/low groups in the TCGA-male cohort (Log-Rank test, p= 0.001).

Q. Survival analyses for circadian risk score high/low groups in the TCGA-female cohort (Log-Rank test, p= 0.02).

R. Survival analyses for circadian risk score high/low groups in the CGGA-male cohort (Log-Rank test, p= 0.001).

S. Survival analyses for circadian risk score high/low groups in the CGGA-female cohort (Log-Rank test, p= 0.0034).

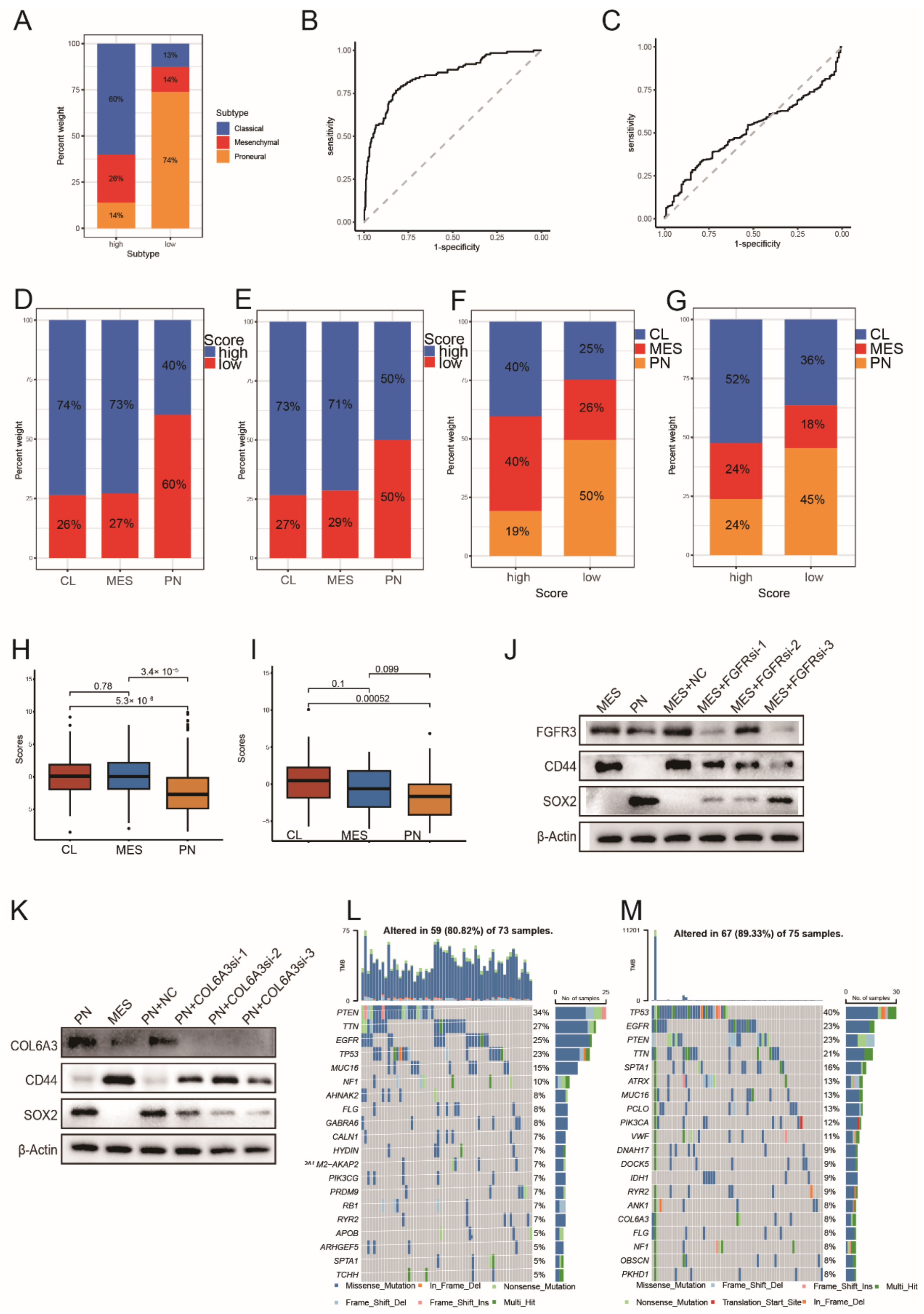

Supplementary Figure S5. Relevance between circadian risk score and molecular profile.

A. The proportion of transcriptome subtypes in circadian risk score high/low groups.

B. ROC curve for PN subtype prediction by circadian risk score in MDSet dataset (AUC=0.85).

C. ROC curve for MES subtype prediction in MDSet dataset (AUC=0.52).

D. The proportion of GBM with high/low risk score in CL, MES and PN subgroups. Male patients in MDSet dataset were analyzed.

E. The proportion of GBM with high/low risk score in CL, MES and PN subgroups. Male patients in MDSet dataset were analyzed.

F. The proportion of transcriptome subtypes in circadian risk score high/low groups analyzed in MDSet-male patients.

G. The proportion of transcriptome subtypes in circadian risk score high/low groups analyzed in MDSet-female patients.

H. The risk score for CL, MES and PN GBM were compared in male patients from MDSet dataset.

I. The risk score for CL, MES and PN GBM were compared in female patients from MDSet dataset.

J. Western blot for MES GBM cells (GBM 267) and PN GBM cells (GBM 8-11) transfected with si-FGFR3. *n*=3.

K. Western blot for MES GBM cells (GBM 267) and PN GBM cells (GBM 8-11) transfected with si-COL6A3. *n*=3.

L. Top 20 most frequently mutant genes in GBM with high risk score.

M. Top 20 most frequently mutant genes in GBM with low risk score.

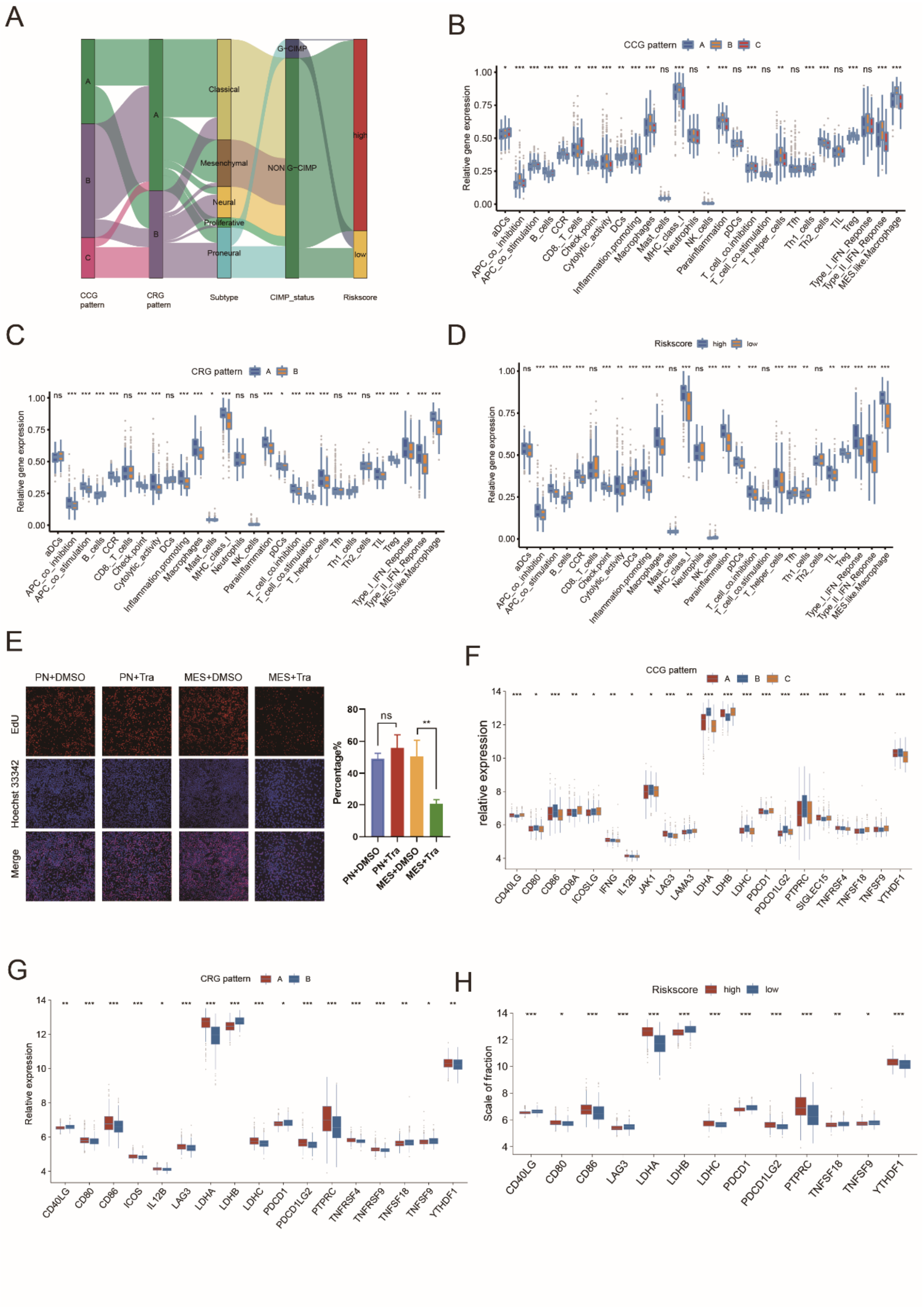

Supplementary Figure S6. Comparison of CCG Patterns, CRG Patterns, Circadian Risk Score, and Their Association with Fundamental Characteristics of GBM.

A. Alluvial diagram of circadian gene patterns in groups with different transcriptome subtypes, G-CIMP status, circadian-related gene patterns and circadian risk score.

B. ssGSEA for immune cell infiltration in circadian core-gene patterns A, B and C (Kruskal-Wallis test, *$p < 0.05$; **$p < 0.01$; ***$p < 0.001$; ns, not significant).

C. ssGSEA for immune cell infiltration in circadian-related gene pattern A and B (Kruskal-Wallis test, *$p < 0.05$; **$p < 0.01$; ***$p < 0.001$; ns, not significant).

D. ssGSEA for immune cell infiltration in circadian risk score high/low groups (Kruskal-Wallis test, *$p < 0.05$; **$p < 0.01$; ***$p < 0.001$; ns, not significant).

E. PN GBM and MES GBM cells pretreated by Trametinib (Tra) and cell viability were detected by EdU assay. The analysis for EdU positive cells were shown in the histogram. (One-way ANOVA, n=3, **$p < 0.01$; ns, not significant)

F. Relative expression of immune check points among circadian core-gene patterns A, B and C (Kruskal-Wallis test, *$p < 0.05$; **$p < 0.01$; ***$p < 0.001$).

G. Relative expression of immune check points in circadian-related gene pattern A and B (Kruskal-Wallis test, *$p < 0.05$; **$p < 0.01$; ***$p < 0.001$).

H Relative expression of immune check points in circadian risk score high/low groups (Kruskal-Wallis test, *$p < 0.05$; **$p < 0.01$; ***$p < 0.001$).

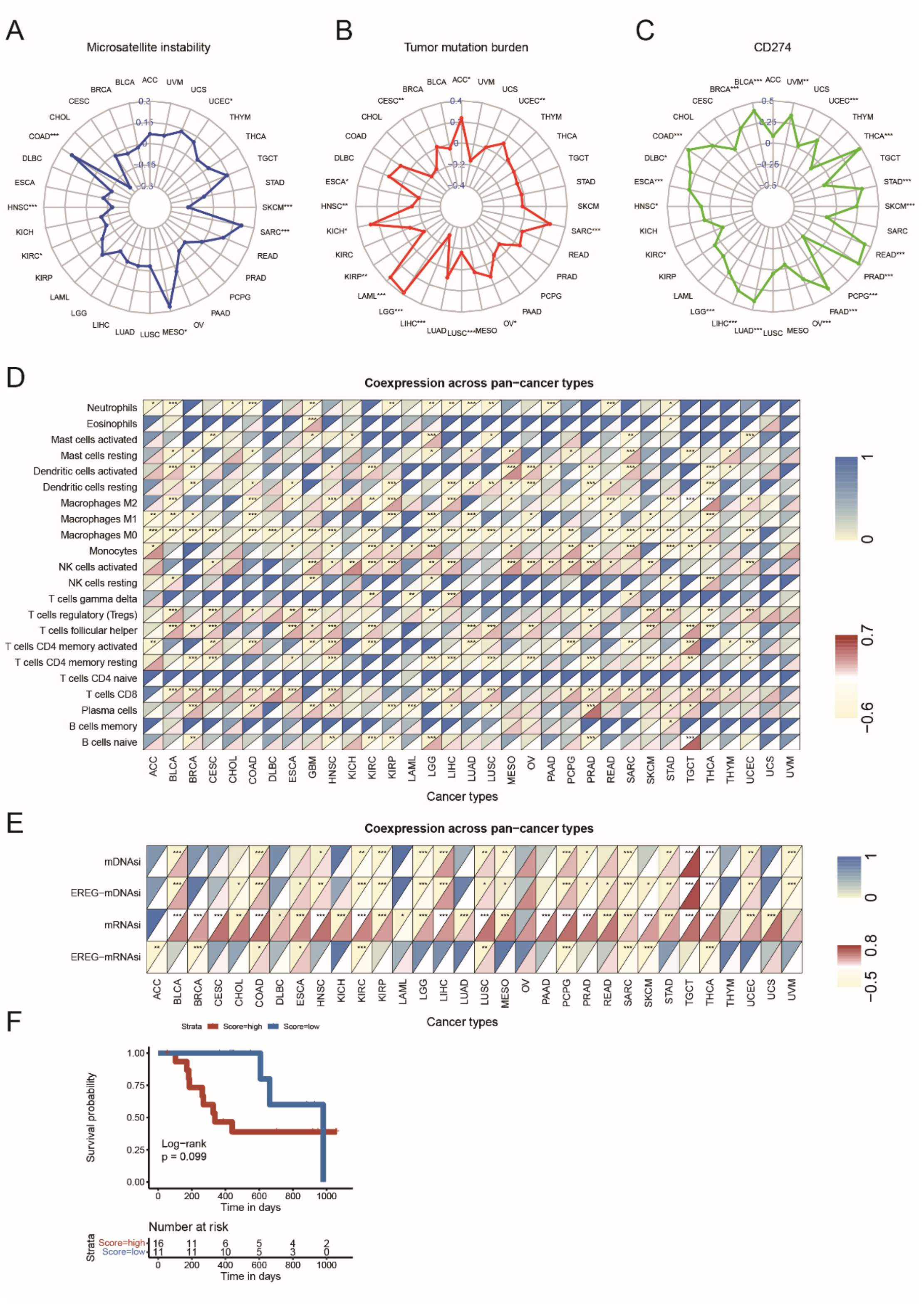

Supplementary Figure S7. The relationship between circadian risk score efficacy of immunotherapy.

A-C. Radar chart of the correlation between circadian risk score and microsatellite

instability (A), tumor mutation burden (B), and PD-L1 expression value (C). The dots in the radar chart represent the R-value of correlation: $R > 0$, positive correlation; and $R < 0$, negative correlation.

D. Correlations between the circadian risk score and immune cell fractions for each cancer type (Pearson test, *$p < 0.05$; **$p < 0.01$; ***$p < 0.001$).

E. Correlation between the circadian risk score and stemness indices for each cancer type. The upper part of each grid showed the P value, and the bottom part showed the correlation coefficient. The asterisks represented the statistical P value. (Pearson test, *$p < 0.05$; **$p < 0.01$; ***$p < 0.001$).

F. Survival analyses for circadian risk score high/low groups in the anti-PD-1 cohort (GSE78220, Log-Rank test, $p = 0.099$).
